# Potential mechanisms involved in regulating muscle protein turnover after acute exercise: A brief review

**DOI:** 10.3389/fphys.2022.1106425

**Published:** 2023-01-09

**Authors:** Guy Hajj-Boutros, Antony D. Karelis, Marina Cefis, José A. Morais, Juliette Casgrain, Gilles Gouspillou, Vita Sonjak

**Affiliations:** ^1^ Research Institute of the McGill University Health Center (MUHC), Montreal, QC, Canada; ^2^ Department of Exercise Science, Université du Québec à Montréal, Montreal, QC, Canada; ^3^ Division of Geriatric Medicine, McGill University, Montreal, QC, Canada

**Keywords:** muscle protein synthesis, muscle protein breakdown, mechanical stress, resistance training, high intensity interval training (HIIT)

## Abstract

It is well established that resistance training increases muscle mass. Indeed, there is evidence to suggest that a single session of resistance training is associated with an increase in muscle protein synthesis in young adults. However, the fundamental mechanisms that are involved in regulating muscle protein turnover rates after an acute bout of physical exercise are unclear. Therefore, this review will briefly focus on summarizing the potential mechanisms behind the growth of skeletal muscle after physical exercise. We also present mechanistic differences that may exist between young and older individuals during muscle protein synthesis and breakdown after physical exercise. Pathways leading to the activation of AKT/mTOR signals after resistance exercise and the activation of AMPK signaling pathway following a HIIT (High intensity interval training) are discussed.

## Introduction

Skeletal muscle atrophy is characterized by a net negative protein balance, while muscle growth results from a positive protein yield (Carbone and Pasiakos, 2019). Resistance exercise (RE) is the most often used intervention to increase muscle size (Borde et al., 2015; Benito et al., 2020). Current guidelines for physical activity by the American College of Sports Medicine and the American Heart Association recommend performing muscle-strengthening activities at least twice a week during non-consecutive days. Indeed, several studies have reported that an acute bout of RE increases muscle protein synthesis in young adults (Brook et al., 2016; Damas et al., 2016). However, the mechanisms that are involved in muscle protein turnover rates after physical exercise are still poorly understood. Therefore, the purpose of this brief review will be to summarize the potential mechanisms behind the growth of skeletal muscle after physical exercise. That is, we will briefly focus on the acute effect of RE and high intensity interval training (HIIT) on muscle protein synthesis and muscle protein breakdown. The present brief review will discuss both of these types of exercises with a focus on their impact on key pathways of skeletal muscle adaption. We will also bring new insight to the literature by identifying different mechanistic pathways of muscle protein turnover rates after physical exercise between young and older individuals. A literature search was conducted on the PubMed database using the following keywords: Physical Activity or Acute Exercise or High Intensity Interval Training or Resistance Training or Muscle Protein Synthesis or Muscle Protein Breakdown or Muscle Protein Turnover AND Young Adults or Older Adult or Aging or Seniors. All languages and article types were included in the search in order to maximize the number of articles found. The reference lists of all the articles found were assessed in order to identify other articles not found during the literature search. No other exclusion criteria were used.

## Skeletal muscle adaptation after acute resistance exercise

Skeletal muscle is highly adaptive tissue and sensitive to various stimuli, such as mechanical stress (e.g., exercise) (Petrella et al., 2006). It is well established that RE represents a mechanical stressor to the skeletal muscle, which initiates muscle hypertrophy by activating anabolic signaling pathways and consequently increases muscle protein synthesis (MPS) (Williamson et al., 2003; Cunningham et al., 2007). By activating MPS, muscle mass increase, improving muscle strength and functional capacity especially in the older population (Benito et al., 2020). Hence, muscle growth is a slow process as rates of protein synthesis needs to exceed rates of protein breakdown. Although RE is a well-known stimulus for muscle hypertrophy in younger adults (Miller et al., 2014), various studies in older adults (65 years and above) found that the fractional synthetic rate (FSR) of muscle protein is diminished following an acute bout of RE (Mayhew et al., 2009; Fry and Rasmussen, 2011). That is, unlike young adults, studies have shown that older adults are not able to increase muscle protein FSR 3 h post-exercise (Paddon-Jones et al., 2004) or even 24 h after an acute RE bout (Mayhew et al., 2009; Fry et al., 2011). Interestingly, it has been noted that baseline fasting FSR does not differ between the age groups suggesting that the response to acute RE in older adults is attenuated, which may be due to the inability of the muscle to increase the stimulation of MPS (Mayhew et al., 2009). A recent study assessed specifically myofibrillar protein synthesis (myoPS) over a 10-week RE and found that myoPS after the first bout of RE was primarily directed towards exercise-induced muscle damage (Damas et al., 2016). RE-induced myoPS and muscle damage seem to decrease throughout the RE program and only in the later phases of RE the myoPS is more likely to contribute to muscle hypertrophy (Damas et al., 2016). myoPS is a more detailed version of the synthesis rates occurred in the muscle and measured from muscle biopsy tissue. Another important factor is the initial fitness level of participants. It has been shown that in participants who engage in resistance and endurance exercise on a daily basis, the muscle protein FSR does not increase after an acute bout of RE to the same extend as in untrained individuals (Damas et al., 2016). The latter notion is in accordance with a finding that acute RE induced muscle damage is greater in untrained than in trained individuals (Gibala et al., 2000).

### Muscle protein synthesis

**FIGURE 1:**
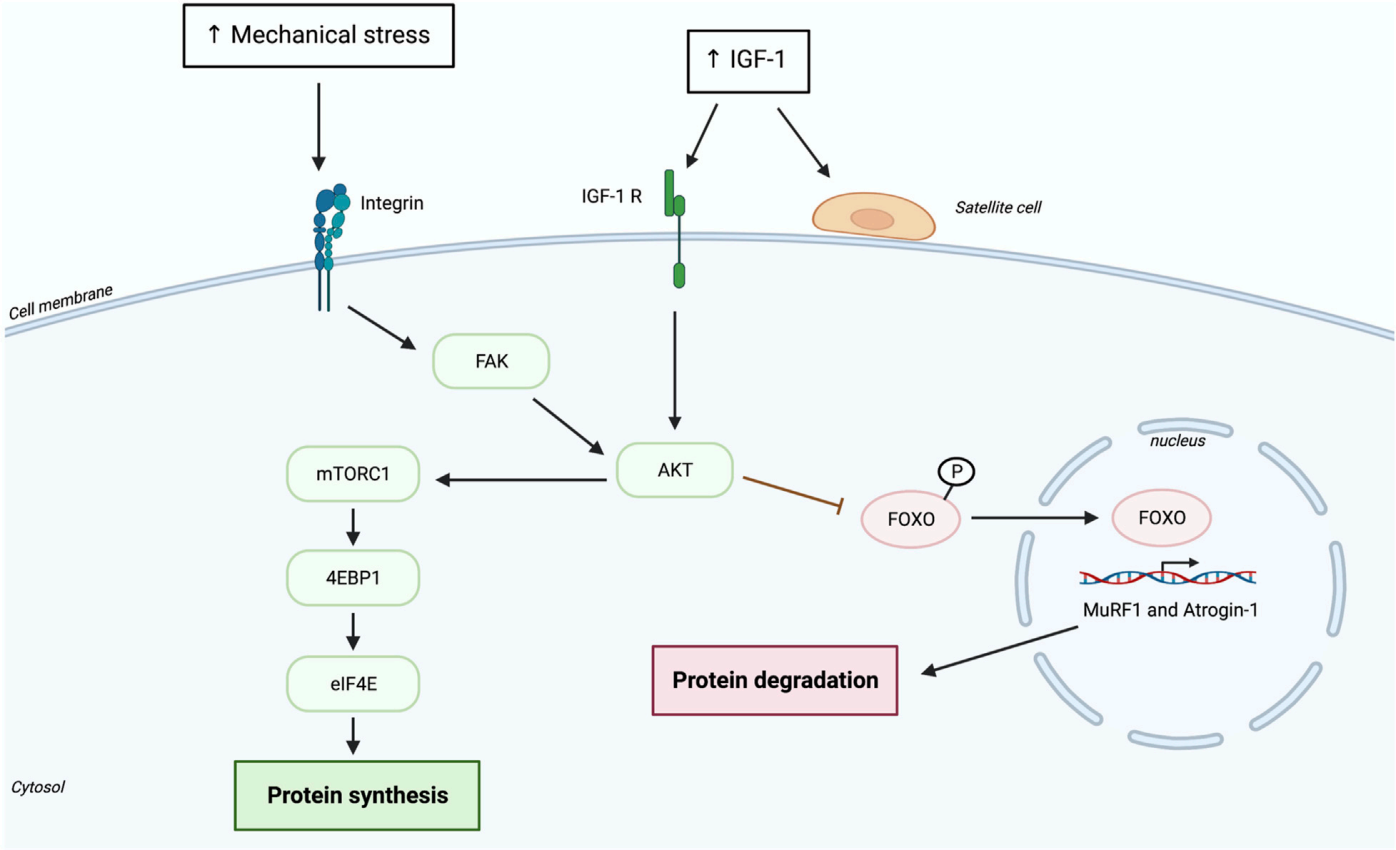
Pathways leading to the activation of AKT/mTOR signals after resistance exercise.

The key pathway regulating protein synthesis is the mammalian target of rapamycin complex 1 (mTORC1) pathway (Figure 1) (Miyazaki et al., 2011). The activity of mTORC1 can be controlled by different factors, such as mechanical signaling (exercise), growth factors [e.g., insulin-like growth factor (IGF-1)] and amino acid availability. IGF-1 seems to have a long-term positive impact on muscle mass and strength by increasing in the muscle and blood circulation following 10 weeks of resistance training in frail older adults (Signh et al., 1999). In addition, acute resistance exercise seems to attenuate mRNA IGF-1 expression by increasing up to 62% following only eight sets of squats in young adults (Bamman et al., 2001). IGF-1 seems to aslo increase in blood circulation during and right after explosive resistance training in young men and women) (Nindl B.C et al., 2012). Results shows that the increase is instant in total IGF-I (pre-exercise = 546 ± 42, mid-exercise = 585 ± 43, post-exercise = 597 ± 45, *p* < .05) (Nindl et al., 2012). It was also reported that acute RE results in an increase in the local expression of IGF-1, well known to activate Akt and mTORC1 (Bamman et al., 2001). However, the exact upstream factors leading to mTORC1 activation in response to mechanical stimulation (e.g., RE) are still partly understood. It has been suggested that there is a mechanosensitive signaling pathway likely involving integrins and acting through phosphatidic acid (PA) and focal adhesion kinase (FAK) that can activate mTORC1 after RE (Brook et al., 2016). The main downstream targets of activated mTORC1 are ribosomal protein s6p70 kinase (S6K1) and eukaryotic initiation factor 4E-binding protein (4E-BP1), which further regulate protein synthesis and ribosomal biogenesis (Koopman et al., 2006; Camera et al., 2016; Chaillou, 2017). Some studies demonstrated that within 1 h, 4 h (Kumar et al., 2009), or even 24 h after an acute RE training older adults do not exhibit any changes in mTORC1, S6K1 and 4E-BP1 phosphorylation, whereas their younger counterparts display an increase in mTOR activation already 3 h after RE, and an increase in phosphorylation of S6K1 and 4E-BP1 6 h after an acute RE bout (Fry et al., 2011). The reason for the diminished or delayed protein synthesis after an acute bout of RE in older adults is not clear, some studies suggest attenuated mTOR phosphorylation and its downstream targets S6K1 and 4E-BP1 (Kumar et al., 2009; Fry et al., 2011), whereas others do not (Drummond et al., 2008; Mayhew et al., 2009). However, only young participants showed a positive correlation between S6K1 phosphorylation and myofibrillar protein synthesis (Kumar et al., 2009). The discrepancy between studies may depend on the structure of the experimental protocol as well as the assessment of the type of protein synthesized (e.g., mixed or myofibrillar) (Brook et al., 2016). Another molecule that is activated likely through an integrin dependent pathway after an acute bout of RE and could be potentially involved in MPS is extracellular signal-regulated kinase (ERK1/2) (Moore et al., 2011; Callahan et al., 2014). ERK1/2 can activate (increase in phosphorylation) the eukaryote initiation factor 4E (eIF4E) independently of mTORC1 *via* its downstream target mitogen-activating protein kinase-interacting kinase 1 (MNK1) (Slack, 2017). ERK1/2 can also potentially activate ribosomal protein S6 (rpS6) through p90 ribosomal S6 kinase (het Veld et al., 2015) as well as directly promote mTORC1 activity (Miyazaki et al., 2011; Fry et al., 2013; Slack, 2017). A study in older adults showed that although mTORC1 was activated after an acute bout of RE, the expression of ERK1/2 was not (Drummond et al., 2008; Williamson et al., 2010). They postulated that delayed protein synthesis seen in the older adults may be due to inadequate stimulation of both pathways concurrently (Drummond et al., 2008) and that perhaps ERK1/2 is involved in the later phase of RE. Although older people do not show any changes in MPS as a response to acute RE (Drummond et al., 2008; Mayhew et al., 2009; Stec et al., 2016), they do present muscle hypertrophy after chronic RE, suggesting that muscle mass gain in the aged muscle requires longer time-period of RE due to reduced sensitivity to mechanical stimuli (Bamman et al., 2007; Mayhew et al., 2009; Li et al., 2012). These studies propose that attenuated protein synthetic response to repeated bouts of RE may contribute to the altered adaptation of the aging muscle (Damas et al., 2016; Stec et al., 2016). It should be noted that increased protein synthesis also depends on the translational efficiency, which relies on translational capacity. The translational capacity of myofibers is mainly determined by the quantity of ribosomes. However, it is important to note that increased transcription does not always lead to successful protein synthesis as alterations in post-transcriptional modification can negatively affect protein abundance in the cell (Kosek et al., 2006). The latter notion goes along with findings in older adults, where diminished FSR response to unaccustomed acute RE was not in accordance with the translation initiation signaling pathways, which was not affected by age (Mayhew et al., 2009), suggesting that muscle protein FSR may be regulated post-transcriptionally (Phillips et al., 1997). It has been suggested that RE can increase translational capacity by affecting ribosome biogenesis where increased number of ribosomes can facilitate mRNA translation and expedite myofiber hypertrophy (Cartee et al., 2016; Stec et al., 2016). Indeed, recent evidence demonstrated that the degree to which myofiber can hypertrophy seems to be related to translation capacity (e.g., ribosomal quantity) (Figueiredo et al., 2015). Older subjects are proposed to have reduced ability to activate ribosome biogenesis following RE (Stec et al., 2016). A study showed that 24 h after first bout of RE aging muscle did not exhibit increased expression of precursor rRNA and ribosomal protein as seen in the young, despite that protein expression levels did not differ between the groups at baseline (Stec et al., 2016). Although aging muscle appears to accrue ribosomes, it is not known if they are functionally intact (Stec et al., 2016), which would consequently impact translational capacity. These findings suggest that attenuated ribosome biogenesis in older subject may be a potential mechanism contributing to blunted hypertrophy response to RE in the aged muscle (Stec et al., 2016). Thus, since there is no need to increased rDNA template, this may further limit the addition of new myonuclei to the myofiber (Cartee et al., 2016). Nevertheless, not all old subjects have the same hypertrophic response to RE. Some older subjects have a better hypertrophic response to the first bout of RE, where the rate of translational initiation is increased leading to elevated translational efficiency (Wagenmakers et al., 2006; Mayhew et al., 2009). This finding is in accordance with increased ribosome protein synthesis that appears within 24 h of RE (Stec et al., 2016). The need for more ribosome protein synthesis may drive the addition of new monnuclei. Thus, mechanotranduction and IGF-I are known to activate satellite cells for myonuclear accretion. However, it is important to mention that only one bout of exercise is unlikely to increase the number myonuclei by much (Burkholder, 2007). Although MPS does not correlate with hypertrophy after the initial bout of RE, the increased ribosomal biogenesis seen at an early stage of RE in high responders may be important to maximize the long-term RE induced hypertrophy (Kim et al., 2007). The delayed hypertrophic response to RE may also depend on the translational capacity of an individual, where some people may need numerous bouts of RE to reach the same mRNA content as individuals who greatly increase mRNA content already after the first bout of RE (Stec et al., 2016). It needs to be also noted that the time point at which muscle biopsy is taken after RE or throughout a time-course of RE represents an important factor as protein and mRNA expression are post-exercise time dependent (Stec et al., 2016).

### Muscle protein breakdown

RE can also lead to protein degradation, especially within the first few hours after an acute bout of RE (Biolo et al., 1995). A study showed that protein fractional breakdown rate (FBR) peaked 3 h post-exercise and gradually decreased to resting rates within 48 h (Phillips et al., 1997). In the latter study there was a positive correlation between FSR and FBR, the net protein balance was negative, which may be due to the fasted state of the participants (Phillips et al., 1997). Although protein FSR has been shown to be impaired in old age, FBR seems not to change with increasing age (Fry et al., 2013). It has been further postulated that protein FBR, which represents a source of amino acids (AA) for FSR in fasted state, may be reduced and hence no change in protein FSR was observed (Phillips et al., 1997). Protein breakdown is regulated through the ubiquitin proteasome system (UPS), where specific E3 ubiquitin ligases, Atrogin-1 and Muscle-RING-finger protein 1 (MuRF1), which are regulated by transcription factor Forkhead box O (FoxO), ubiquitinate a targeted protein for degradation (Raue et al., 2007; Abruzzo et al., 2010; Pugh et al., 2015). Interestingly, both MuRF1 and Atrogin-1 mRNA have been shown to be upregulated in octogenarian women, while young women only displayed an increase in MuRF1 mRNA expression after an acute bout of RE (Raue et al., 2007). This finding suggests that very old muscle (>80 years of age) has a greater proteolytic response, which may promote protein breakdown after RE (Raue et al., 2007; Williamson et al., 2010) and suggest the presence of age-related alterations of the protein kinase B (Akt) pathway, which phosphorylation may be insufficient to suppresses FoxO during an anabolic stimuli (Raue et al., 2007; Williamson et al., 2010; Fry et al., 2013). Other studies additionally suggested that the activation of the UPS may be required for the repairment of contractile proteins after an acute bout of RE-induced muscle damage (Borgenvik et al., 2012; Damas et al., 2016). However, despite evidence that the aging muscle has a higher proteolytic rate (Pennings et al., 2012), in advanced age (>80 years of age) the ubiquitin-proteasome mediated gene expression appears to be down regulated (Cuthbertson et al., 2005; Casperson et al., 2012). This could occur due to alterations in translational capacity (e.g., ribosomal quantity and quality) or due to alterations in post-translational modifications that affect protein structure and function. A study in octogenarian women showed that older inactive women have an increased expression of FOXO3A and MuRF1, but with no change in Atrogin-1 when compared to young controls (Cuthbertson et al., 2005). Another important pathway involved in MPB is the autophagosomal-lysosomal system. It has been demonstrated that in older people after an acute bout of RE, markers of autophagy are reduced (Glynn et al., 2010; Fry et al., 2013). Microtubule-associated protein light chain 3 (LC3) is a protein used to monitor autophagy. It has been widely used to monitor the number of autophagosomes as well as autophagic activity. A study showed that the expression of cytosolic microtubule-associated protein 1 light chain 3 (LC3B-I) did not change after exercise, while the autophagosomal membrane-associated form (LC3B-II) reduced, furthermore, the conversion of LC3B-I to LCB3-II also decreased (Glynn et al., 2010; Fry et al., 2013). This suggests that the autophagic response is reduced after an acute bout of RE in older individuals, which may contribute to the accumulation of misfolded and damaged proteins (Fry et al., 2013).

### High intensity interval training (HIIT)

Maintenance of optimal muscle power and cardiorespiratory fitness (CRF) are the most effective prevention strategies that individuals can engage in during advancing age (Karlsen et al., 2017). Exercise intensity levels appear to be an important determinant of muscular adaptation to the selected physical activity (e.g., resistance exercise or endurance exercise). Because of the positive effects of aerobic high intensity training on CRF leading to reduced risks for cardiovascular disease (Karlsen et al., 2017), a new type of exercise program called high intensity interval training (HIIT) has gained a lot of attention (Boutros et al., 2019).

**FIGURE 2:**
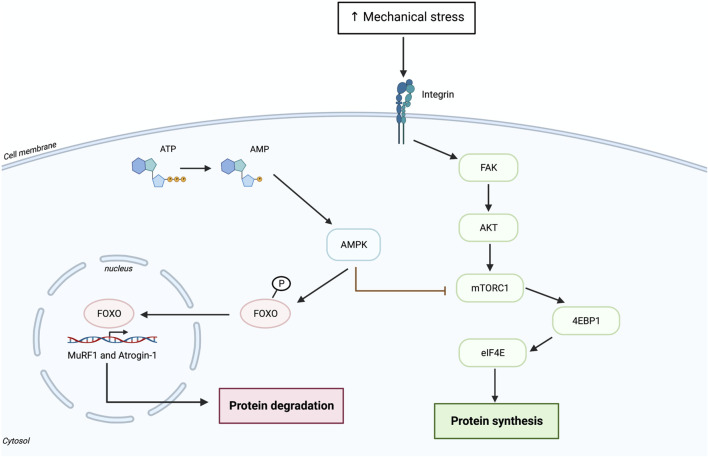
Activation of AMPK signaling pathway following aerobic exercise.

HIIT is a time-efficient training method characterized by repeated bouts of short aerobic activity at high intensities (e.g., 85%–95% of maximal heart rate or 80%–90% of VO_2_ max) with short resting periods or low intensity bouts allowing recovery (Karlsen et al., 2017; Robinson et al., 2017). HIIT is suggested to combine some parts of endurance exercise (EE) and RE as it activates aerobic adaptation and involves high intensity muscle contraction (Bell et al., 2015). Therefore, because of the marked loss of muscle mass and strength in the older adult population, HIIT should be supplemented with RE with a focus on lower limbs. However, different physical demands from RE and EE result in different muscular and cellular adaptations. As previously described, RE induces the activation of mTORC1 pathways, which leads to increased protein synthesis and eventually to muscle hypertrophy. Conversely, EE activates AMPK, which induces the expression of PGC1-α and consequent regulation of oxidative metabolism and mitochondrial biogenesis. AMPK is a negative regulator of mTORC1 and an activator of the FOXO transcription factor family which govern in large part MPB (Figure 2). It is important to note that AMPK activation is one many pathways leading to PGC-1a activation upon EE. Calcineurin activation by prolonged cytosolic calcium oscillations is likely as important as AMPK activation leading to the activation of PGC-1a. Some (Hawley, 2009; Farup et al., 2012), but not all (Apró et al., 2013; Murach and Bagley, 2016) studies showed that the combination of traditional EE and RE has a negative impact on muscle adaptation, where AMPK activation after EE interferes with the activation of mTORC1 pathway induced by RE. Hence, the timing of each type of exercise is important to gain positive effects/adaptations induced by both RE and EE (Murach and Bagley, 2016). Interestingly, acute bout of RE has shown to increase AMPK expression, but only in untrained subjects (Drummond et al., 2008; Coffey and Hawley, 2017). In addition, older muscle after a single bout of RE seems to exhibit greater levels of AMPK, which may contribute to the delayed protein synthesis (Drummond et al., 2008). A study in younger adults showed that acute HIIT followed immediately after RE does not interfere with RE-related adaptations (Pugh et al., 2015). They found that markers of mitochondrial biogenesis were greatly increased in the RE + HIIT program compared to only RE. However, they did not find any change in the expression of signaling molecules downstream of mTORC1 (e.g., S6K1 and 4E-BP1) (Pugh et al., 2015), suggesting attenuated protein synthesis. Another study in older adults separately assessed the impact of acute RE and HIIT on MPS (Bell et al., 2015). They found that HIIT significantly increases MPS although to a lesser extend as RE. Interestingly, only HIIT seemed to stimulate sarcoplasmic protein synthesis 24 h after exercise and it has been contemplated that increased mitochondrial protein synthesis may contribute to the high sarcoplasmic protein fractional synthesis rate (FSR) (Bell et al., 2015). Indeed, 12 weeks of HIIT and RE in older men resulted in increased mitochondrial FSR as well as mitochondrial biogenesis and consequently increased mitochondrial oxidative capacity and improved CRF (Robinson et al., 2017). However, increased muscle strength was noticed only after RE and not in HIIT (Robinson et al., 2017). The later finding may be due to the short recovery period in older men after each HIIT session, which may attenuate development of muscle strength (Herbert et al., 2015). From the former study, it appears that HIIT may induce muscle hypertrophy to a certain degree in older adults by potentially inducing similar muscle damage as RE and stimulate MPS (Schoenfeld, 2012; Bell et al., 2015; Damas et al., 2016). Another study in octogenarian men that combined RE and HIIT (RE + HIIT) for 9 weeks showed improved muscle strength, functional capacities (e.g., walking speed) and aerobic capacity (Guadalupe-Grau et al., 2017). Nonetheless, future studies should assess the effect of acute RE + HIIT and long-term RE + HIIT on alterations in muscle mass and muscle protein synthesis in older adults as well as the degree to which HIIT may attenuate adaptive responses induced by RE. The combination of RE and HIIT in a single session seem to serve as a feasible and time effective exercise regime for older adults. However, there is still lack of consensus about the intensity and duration that should be applied to very old individuals (>80 years of age). The increased loss of muscle mass and strength in the older adult population could limit exercise at high intensity, especially in the case of frail older individuals or very old participants who are at a higher risk of falls. Therefore, the structure of HIIT should be tailored to the individual’s needs. Indeed, it is important to give them enough time to recover between intervals so that they are able to perform the exercise session in a safe manner. Given the extreme exercise regime of HIIT it is doubtful that the older adult population could safely or practically adopt this type of exercise, hence, proper intensity level and supervision of the training program are important factors contributing to reach maximal benefits from HIIT.

Even though resistance and endurance exercise training induce numerous positive effects, the adaptation processes vary between individuals. It is therefore important to consider the initial fitness level as untrained individuals have a greater capacity to initiate molecular signaling after physical activity (Coffey and Hawley, 2017). Age also has a great importance on the adaptations following acute exercise (Table 1). Indeed, the latter was demonstrated by findings where acute bouts of RE induced AMPK phosphorylation (Dreyer et al., 2006; Koopman et al., 2006) and EE activated mTOR signaling (Wilkinson et al., 2008).

## Conclusion

Acute physical exercise, in particular RE, has been shown to increase MPS. However, the mechanisms that are involved in muscle protein turnover rates after acute physical exercise are still poorly understood. Indeed, a key pathway in regulating muscle protein synthesis and breakdown is the mammalian target of rapamycin complex 1 (mTORC1) pathway. The activation of AMPK signaling pathway also seem to be implicated. However, the mechanistic pathways seem to differ between older and younger individuals. Furthermore, even though RE induces numerous positive effects, the adaptation processes vary between individuals. It is important to consider clinical characteristics of the targeted group including age and initial fitness level as untrained individuals have a greater capacity to initiate molecular signaling after the initial bout of RE (Coffey and Hawley, 2017). However, it is still difficult to predict that everyone will respond similarly to a given type of exercise, especially in the older population where adaptational processes get altered and the response to exercise becomes even more complex.

**TABLE 1:** Activated protein following acute resistance exercise.

Activated protein following acute 1 resistance exercise	Young	Old
Muscle protein synthesis		
mTORC1	↑↑↑	↑
ERK1/2	↑↑↑	↔
S6K1	↑↑↑	↑
Ribosomal protein	↑↑↑	↑
Muscle protein breakdown		
MuRF1	↑↑	↑↑↑
Atrogin-1	↔	↑↑
LC3B-1	NS	↓
AMPK	↑	↑↑↑

## References

[B1] Abruzzo P. M. Di Tullio S. Marchionni C. Belia S. Fanó G. Zampieri S. (2010). Oxidative stress in the denervated muscle. Free Radic. Res. 44 (5), 563–576. 10.3109/10715761003692487 20298122

[B2] Apró W. Wang L. Pontén M. Blomstrand E. Sahlin K. (2013). Resistance exercise induced mTORC1 signaling is not impaired by subsequent endurance exercise in human skeletal muscle. Am. J. Physiology-Endocrinology Metabolism 305 (1), E22–E32. 10.1152/ajpendo.00091.2013 23632629

[B3] Bamman M. M. Petrella J. K. Kim J.-s. Mayhew D. L. Cross J. M. (2007). Cluster analysis tests the importance of myogenic gene expression during myofiber hypertrophy in humans. J. Appl. physiology 102, 2232–2239. 10.1152/japplphysiol.00024.2007 17395765

[B4] Bamman M. M. Shipp J. R. Jiang J. Gower B. A. Hunter G. R. Goodman A. (2001). Mechanical load increases muscle IGF-I and androgen receptor mRNA concentrations in humans. Am. J. physiology-endocrinology metabolism 280 (3), E383–E390. 10.1152/ajpendo.2001.280.3.E383 11171591

[B5] Bell K. E. Séguin C. Parise G. Baker S. K. Phillips S. M. (2015). Day-to-day changes in muscle protein synthesis in recovery from resistance, aerobic, and high-intensity interval exercise in older men. Journals Gerontology Ser. A 70 (8), 1024–1029. 10.1093/gerona/glu313 25650305

[B6] Benito P. J. Cupeiro R. Ramos-Campo D. J. Alcaraz P. E. Rubio-Arias J. Á. (2020). A systematic review with meta-analysis of the effect of resistance training on whole-body muscle growth in healthy adult males. Int. J. Environ. Res. public health 17 (4), 1285. 10.3390/ijerph17041285 32079265 PMC7068252

[B7] Biolo G. Maggi S. P. Williams B. D. Tipton K. D. Wolfe R. R. (1995). Increased rates of muscle protein turnover and amino acid transport after resistance exercise in humans. Am. J. Physiol. 268 (3), E514–E520. 10.1152/ajpendo.1995.268.3.E514 7900797

[B8] Borde R. Hortobágyi T. Granacher U. (2015). Dose–response relationships of resistance training in healthy old adults: A systematic review and meta-analysis. Sports Med. 45 (12), 1693–1720. 10.1007/s40279-015-0385-9 26420238 PMC4656698

[B9] Borgenvik M. Apro W. Blomstrand E. (2012). Intake of branched-chain amino acids influences the levels of MAFbx mRNA and MuRF-1 total protein in resting and exercising human muscle. Am. J. Physiol. Endocrinol. Metab. 302 (5), E510–E521. 10.1152/ajpendo.00353.2011 22127230

[B10] Boutros G. E. H. Morais J. A. Karelis A. D. (2019). Current concepts in healthy aging and physical activity: A viewpoint. J. Aging Phys. Activity 27 (5), 755–761. 10.1123/japa.2018-0208 30747553

[B11] Brook M. Wilkinson D. Phillips B. Perez Schindler J. Philp A. Smith K. (2016). Skeletal muscle homeostasis and plasticity in youth and ageing: Impact of nutrition and exercise. Acta physiol. 216 (1), 15–41. 10.1111/apha.12532 PMC4843955 26010896

[B12] Burkholder T. J. (2007). Mechanotransduction in skeletal muscle. Front. Bioscience-Landmark 12 (1), 174–191. 10.2741/2057 PMC2043154 17127292

[B13] Callahan D. M. Bedrin N. G. Subramanian M. Berking J. Ades P. A. Toth M. J. (2014). Age-related structural alterations in human skeletal muscle fibers and mitochondria are sex specific: Relationship to single-fiber function. J. Appl. physiology 116 (12), 1582–1592. 10.1152/japplphysiol.01362.2013 PMC4064376 24790014

[B14] Camera D. M. Smiles W. J. Hawley J. A. (2016). Exercise-induced skeletal muscle signaling pathways and human athletic performance. Free Radic. Biol. Med. 98, 131–143. 10.1016/j.freeradbiomed.2016.02.007 26876650

[B15] Carbone J. W. Pasiakos S. M. (2019). Dietary protein and muscle mass: Translating science to application and health benefit. Nutrients 11 (5), 1136. 10.3390/nu11051136 31121843 PMC6566799

[B16] Cartee G. D. Hepple R. T. Bamman M. M. Zierath J. R. (2016). Exercise promotes healthy aging of skeletal muscle. Cell metab. 23 (6), 1034–1047. 10.1016/j.cmet.2016.05.007 27304505 PMC5045036

[B17] Casperson S. L. Sheffield-Moore M. Hewlings S. J. Paddon-Jones D. (2012). Leucine supplementation chronically improves muscle protein synthesis in older adults consuming the RDA for protein. Clin. Nutr. 31 (4), 512–519. 10.1016/j.clnu.2012.01.005 22357161 PMC3640444

[B18] Chaillou T. (2017). Impaired ribosome biogenesis could contribute to anabolic resistance to strength exercise in the elderly. J. physiology 595 (5), 1447–1448. 10.1113/JP273773 PMC5330918 28105708

[B19] Coffey V. G. Hawley J. A. (2017). Concurrent exercise training: Do opposites distract? J. physiology 595 (9), 2883–2896. 10.1113/JP272270 PMC5407958 27506998

[B20] Cunningham J. T. Rodgers J. T. Arlow D. H. Vazquez F. Mootha V. K. Puigserver P. (2007). mTOR controls mitochondrial oxidative function through a YY1-PGC-1alpha transcriptional complex. nature 450 (7170), 736–740. 10.1038/nature06322 18046414

[B21] Cuthbertson D. Smith K. Babraj J. Leese G. Waddell T. Atherton P. (2005). Anabolic signaling deficits underlie amino acid resistance of wasting, aging muscle. FASEB J. 19 (3), 422–424. 10.1096/fj.04-2640fje 15596483

[B22] Damas F. Phillips S. M. Libardi C. A. Vechin F. C. Lixandrão M. E. Jannig P. R. (2016). Resistance training‐induced changes in integrated myofibrillar protein synthesis are related to hypertrophy only after attenuation of muscle damage. J. physiology 594 (18), 5209–5222. 10.1113/JP272472 PMC5023708 27219125

[B23] Dreyer H. C. Fujita S. Cadenas J. G. Chinkes D. L. Volpi E. Rasmussen B. B. (2006). Resistance exercise increases AMPK activity and reduces 4E‐BP1 phosphorylation and protein synthesis in human skeletal muscle. J. physiology 576 (2), 613–624. 10.1113/jphysiol.2006.113175 PMC1890364 16873412

[B24] Drummond M. J. Dreyer H. C. Pennings B. Fry C. S. Dhanani S. Dillon E. L. (2008). Skeletal muscle protein anabolic response to resistance exercise and essential amino acids is delayed with aging. J. Appl. physiology 104 (5), 1452–1461. 10.1152/japplphysiol.00021.2008 PMC2715298 18323467

[B25] Farup J. Kjølhede T. Sørensen H. Dalgas U. Møller A. B. Vestergaard P. F. (2012). Muscle morphological and strength adaptations to endurance vs. resistance training. J. Strength & Cond. Res. 26 (2), 398–407. 10.1519/JSC.0b013e318225a26f 22266546

[B26] Figueiredo V. C. Caldow M. K. Massie V. Markworth J. F. Cameron-Smith D. Blazevich A. J. (2015). Ribosome biogenesis adaptation in resistance training-induced human skeletal muscle hypertrophy. Am. J. Physiology-Endocrinology And Metabolism 309 (1), E72–E83. 10.1152/ajpendo.00050.2015 25968575

[B27] Fry C. S. Drummond M. J. Glynn E. L. Dickinson J. M. Gundermann D. M. Timmerman K. L. (2011). Aging impairs contraction-induced human skeletal muscle mTORC1 signaling and protein synthesis. Skelet. muscle 1 (1), 11. 10.1186/2044-5040-1-11 21798089 PMC3156634

[B28] Fry C. S. Drummond M. J. Glynn E. L. Dickinson J. M. Gundermann D. M. Timmerman K. L. (2013). Skeletal muscle autophagy and protein breakdown following resistance exercise are similar in younger and older adults. Journals Gerontology Ser. A Biomed. Sci. Med. Sci. 68 (5), 599–607. 10.1093/gerona/gls209 PMC3623482 23089333

[B29] Fry C. S. Rasmussen B. B. (2011). Skeletal muscle protein balance and metabolism in the elderly. Curr. aging Sci. 4 (3), 260–268. 10.2174/1874609811104030260 21529326 PMC5096733

[B30] Gibala M. J. Interisano S. A. Tarnopolsky M. A. Roy B. D. MacDonald J. R. Yarasheski K. E. (2000). Myofibrillar disruption following acute concentric and eccentric resistance exercise in strength-trained men. Can. J. Physiol. Pharmacol. 78 (8), 656–661. 10.1139/y00-036 10958167

[B31] Glynn E. L. Fry C. S. Drummond M. J. Dreyer H. C. Dhanani S. Volpi E. (2010). Muscle protein breakdown has a minor role in the protein anabolic response to essential amino acid and carbohydrate intake following resistance exercise. Am. J. Physiol. Regul. Integr. Comp. Physiol. 299 (2), R533–R540. 10.1152/ajpregu.00077.2010 20519362 PMC2928613

[B32] Guadalupe-Grau A. Aznar-Laín S. Mañas A. Castellanos J. Alcázar J. Ara I. (2017). Short-and long-term effects of concurrent strength and hiit training in octogenarians with COPD. J. aging Phys. activity 25 (1), 105–115. 10.1123/japa.2015-0307 27402660

[B33] Hawley J. A. (2009). Molecular responses to strength and endurance training: Are they incompatible? Appl. physiology, Nutr. metabolism 34 (3), 355–361. 10.1139/H09-023 19448698

[B34] Herbert P. Grace F. M. Sculthorpe N. F. (2015). Exercising caution: Prolonged recovery from a single session of high-intensity interval training in older men. J. Am. Geriatr. Soc. 63 (4), 817–818. 10.1111/jgs.13365 25900496

[B35] het Veld L. P. O. van Rossum E. Kempen G. I. de Vet H. C. Hajema K. Beurskens A. J. (2015). Fried phenotype of frailty: Cross-sectional comparison of three frailty stages on various health domains. BMC Geriatr. 15 (1), 77. 10.1186/s12877-015-0078-0 26155837 PMC4496916

[B36] Karlsen T. Aamot I.-L. Haykowsky M. Rognmo Ø. (2017). High intensity interval training for maximizing health outcomes. Prog. Cardiovasc. Dis. 60 (1), 67–77. 10.1016/j.pcad.2017.03.006 28385556

[B37] Kim J.-s. Petrella J. K. Cross J. M. Bamman M. M. (2007). Load-mediated downregulation of myostatin mRNA is not sufficient to promote myofiber hypertrophy in humans: A cluster analysis. J. Appl. physiology 103 (5), 1488–1495. 10.1152/japplphysiol.01194.2006 17673556

[B38] Koopman R. Zorenc A. H. Gransier R. J. Cameron-Smith D. van Loon L. J. (2006). Increase in S6K1 phosphorylation in human skeletal muscle following resistance exercise occurs mainly in type II muscle fibers. Am. J. Physiology-Endocrinology Metabolism 290 (6), E1245–E1252. 10.1152/ajpendo.00530.2005 16434552

[B39] Kosek D. J. Kim J.-s. Petrella J. K. Cross J. M. Bamman M. M. (2006). Efficacy of 3 days/wk resistance training on myofiber hypertrophy and myogenic mechanisms in young vs. older adults. J. Appl. physiology 101 (2), 531–544. 10.1152/japplphysiol.01474.2005 16614355

[B40] Kumar V. Selby A. Rankin D. Patel R. Atherton P. Hildebrandt W. (2009). Age‐related differences in the dose–response relationship of muscle protein synthesis to resistance exercise in young and old men. J. physiology 587 (1), 211–217. 10.1113/jphysiol.2008.164483 PMC2670034 19001042

[B41] Li M. Verdijk L. B. Sakamoto K. Ely B. Van Loon L. J. Musi N. (2012). Reduced AMPK-ACC and mTOR signaling in muscle from older men, and effect of resistance exercise. Mech. ageing Dev. 133 (11-12), 655–664. 10.1016/j.mad.2012.09.001 23000302 PMC3631591

[B42] Mayhew D. L. Kim J.-s. Cross J. M. Ferrando A. A. Bamman M. M. (2009). Translational signaling responses preceding resistance training-mediated myofiber hypertrophy in young and old humans. J. Appl. physiology 107 (5), 1655–1662. 10.1152/japplphysiol.91234.2008 PMC2777794 19589955

[B43] Miller M. S. Callahan D. M. Toth M. J. (2014). Skeletal muscle myofilament adaptations to aging, disease, and disuse and their effects on whole muscle performance in older adult humans. Front. physiology 5, 369. 10.3389/fphys.2014.00369 PMC4176476 25309456

[B44] Miyazaki M. McCarthy J. J. Fedele M. J. Esser K. A. (2011). Early activation of mTORC1 signalling in response to mechanical overload is independent of phosphoinositide 3‐kinase/Akt signalling. J. physiology 589 (7), 1831–1846. 10.1113/jphysiol.2011.205658 PMC3099033 21300751

[B45] Moore D. Atherton P. Rennie M. Tarnopolsky M. Phillips S. (2011). Resistance exercise enhances mTOR and MAPK signalling in human muscle over that seen at rest after bolus protein ingestion. Acta physiol. 201 (3), 365–372. 10.1111/j.1748-1716.2010.02187.x 20874802

[B46] Murach K. A. Bagley J. R. (2016). Skeletal muscle hypertrophy with concurrent exercise training: Contrary evidence for an interference effect. Sports Med. 46 (8), 1029–1039. 10.1007/s40279-016-0496-y 26932769

[B47] Nindl B. C. Urso M. L. Pierce J. R. Scofield D. E. Barnes B. R. Kraemer W. J. (2012). IGF-I measurement across blood, interstitial fluid, and muscle biocompartments following explosive, high-power exercise. Am. J. Physiology-Regulatory, Integr. Comp. Physiology 303 (10), R1080–R1089. 10.1152/ajpregu.00275.2012 22933025

[B48] Paddon-Jones D. Sheffield-Moore M. Zhang X.-J. Volpi E. Wolf S. E. Aarsland A. (2004). Amino acid ingestion improves muscle protein synthesis in the young and elderly. Am. J. Physiology-Endocrinology And Metabolism 286 (3), E321–E328. 10.1152/ajpendo.00368.2003 14583440

[B49] Pennings B. Groen B. de Lange A. Gijsen A. P. Zorenc A. H. Senden J. M. (2012). Amino acid absorption and subsequent muscle protein accretion following graded intakes of whey protein in elderly men. Am. J. Physiology-Endocrinology Metabolism 302 (8), E992–E999. 10.1152/ajpendo.00517.2011 22338070

[B50] Petrella J. K. Kim J.-s. Cross J. M. Kosek D. J. Bamman M. M. (2006). Efficacy of myonuclear addition may explain differential myofiber growth among resistance-trained young and older men and women. Am. J. Physiology-Endocrinology Metabolism 291 (5), E937–E946. 10.1152/ajpendo.00190.2006 16772322

[B51] Phillips S. M. Tipton K. D. Aarsland A. Wolf S. E. Wolfe R. R. (1997). Mixed muscle protein synthesis and breakdown after resistance exercise in humans. Am. J. Physiol. 273 (1), E99–E107. 10.1152/ajpendo.1997.273.1.E99 9252485

[B52] Pugh J. K. Faulkner S. H. Jackson A. P. King J. A. Nimmo M. A. (2015). Acute molecular responses to concurrent resistance and high‐intensity interval exercise in untrained skeletal muscle. Physiol. Rep. 3 (4), e12364. 10.14814/phy2.12364 25902785 PMC4425969

[B53] Raue U. Slivka D. Jemiolo B. Hollon C. Trappe S. (2007). Proteolytic gene expression differs at rest and after resistance exercise between young and old women. Journals Gerontology Ser. A Biol. Sci. Med. Sci. 62 (12), 1407–1412. 10.1093/gerona/62.12.1407 18166693

[B54] Robinson M. M. Dasari S. Konopka A. R. Johnson M. L. Manjunatha S. Esponda R. R. (2017). Enhanced protein translation underlies improved metabolic and physical adaptations to different exercise training modes in young and old humans. Cell metab. 25 (3), 581–592. 10.1016/j.cmet.2017.02.009 28273480 PMC5423095

[B55] Schoenfeld B. J. (2012). Does exercise-induced muscle damage play a role in skeletal muscle hypertrophy? J. Strength & Cond. Res. 26 (5), 1441–1453. 10.1519/JSC.0b013e31824f207e 22344059

[B56] Singh M. A. F. Ding W. Manfredi T. J. Solares G. S. O’Neill E. F. Clements K. M. (1999). Insulin-like growth factor I in skeletal muscle after weight-lifting exercise in frail elders. Am. J. Physiology-Endocrinology And Metabolism 277 (1), E135–E143. 10.1152/ajpendo.1999.277.1.E135 10409137

[B57] Slack C. (2017). Ras signaling in aging and metabolic regulation. Nutr. healthy aging 4 (3), 195–205. 10.3233/NHA-160021 29276789 PMC5734121

[B58] Stec M. J. Kelly N. A. Many G. M. Windham S. T. Tuggle S. C. Bamman M. M. (2016). Ribosome biogenesis may augment resistance training-induced myofiber hypertrophy and is required for myotube growth *in vitro* . Am. J. Physiol. Endocrinol. Metab. 310 (8), E652–e661. 10.1152/ajpendo.00486.2015 26860985 PMC4835943

[B59] Wagenmakers A. J. Baar K. Nader G. Bodine S. (2006). Resistance exercise, muscle loading/unloading and the control of muscle mass. Essays Biochem. 42, 61–74. 10.1042/bse0420061 17144880

[B60] Wilkinson S. B. Phillips S. M. Atherton P. J. Patel R. Yarasheski K. E. Tarnopolsky M. A. (2008). Differential effects of resistance and endurance exercise in the fed state on signalling molecule phosphorylation and protein synthesis in human muscle. J. physiology 586 (15), 3701–3717. 10.1113/jphysiol.2008.153916 PMC2538832 18556367

[B61] Williamson D. Gallagher P. Harber M. Hollon C. Trappe S. (2003). Mitogen activated protein kinase (MAPK) pathway activation: Effects of age and acute exercise on human skeletal muscle. J. physiology 547 (3), 977–987. 10.1113/jphysiol.2002.036673 PMC2342728 12562918

[B62] Williamson D. L. Raue U. Slivka D. R. Trappe S. (2010). Resistance exercise, skeletal muscle FOXO3A, and 85-year-old women. Journals Gerontology Ser. A Biomed. Sci. Med. Sci. 65 (4), 335–343. 10.1093/gerona/glq005 PMC2844061 20139145

